# DNA Methylation as a Biomarker of Treatment Response Variability in Serious Mental Illnesses: A Systematic Review Focused on Bipolar Disorder, Schizophrenia, and Major Depressive Disorder

**DOI:** 10.3390/ijms19103026

**Published:** 2018-10-04

**Authors:** Charanraj Goud Alladi, Bruno Etain, Frank Bellivier, Cynthia Marie-Claire

**Affiliations:** 1 Department of Pharmacology, Jawaharlal Institute of Postgraduate Medical Education and Research, Puducherry 605006, India; charan.raj002@gmail.com; 2 INSERM U1144 Variabilité de réponse aux psychotropes, Université Paris Descartes, Sorbonne Paris Cité, 75006 Paris, France; Bruno.Etain@inserm.fr (B.E.); frank.bellivier@inserm.fr (F.B.); 3 AP-HP, GH Saint-Louis—Lariboisière—F. Widal, Pôle de Psychiatrie et de Médecine Addictologique, 75475 Paris CEDEX 10, France; 4 Fondation Fondamental, 94000 Créteil, France

**Keywords:** schizophrenia, bipolar disorder, major depressive disorder, DNA methylation, response variability

## Abstract

So far, genetic studies of treatment response in schizophrenia, bipolar disorder, and major depression have returned results with limited clinical utility. A gene × environment interplay has been proposed as a factor influencing not only pathophysiology but also the treatment response. Therefore, epigenetics has emerged as a major field of research to study the treatment of these three disorders. Among the epigenetic marks that can modify gene expression, DNA methylation is the best studied. We performed a systematic search (PubMed) following Preferred Reporting Items for Systematic Reviews and Meta-Analyses (PRISMA guidelines for preclinical and clinical studies focused on genome-wide and gene-specific DNA methylation in the context of schizophrenia, bipolar disorders, and major depressive disorder. Out of the 112 studies initially identified, we selected 31 studies among them, with an emphasis on responses to the gold standard treatments in each disorder. Modulations of DNA methylation levels at specific CpG sites have been documented for all classes of treatments (antipsychotics, mood stabilizers, and antidepressants). The heterogeneity of the models and methodologies used complicate the interpretation of results. Although few studies in each disorder have assessed the potential of DNA methylation as biomarkers of treatment response, data support this hypothesis for antipsychotics, mood stabilizers and antidepressants.

## 1. Introduction

Schizophrenia, bipolar disorder, and major depressive disorder are severe mental illnesses (SMI) defined by classifications such as Diagnostic and Statistical Manual of Mental Disorders (DSM-5) and International Classification of Diseases (ICD10), of sufficient duration to meet diagnostic criteria and resulting in significant functional impairment [1]. Schizophrenia (SCZ), bipolar disorder (BD), and major depressive disorder (MDD) are associated with poor health outcomes, global disability, and public health burden [2,3]. While SCZ affects 1% of the population [4], 2% of the population is affected by BD worldwide [5]. Depression is the second leading cause of global health problem with 6.6–21% of the population in high-income countries and 6.5–18.4% of the population in low–middle-income countries prone to succumb this disorder [6,7]. Briefly, SCZ, BD, and MDD are characterized by acute episodes including psychotic, depressive, and/or manic features that are superimposed, in some patients, with a chronic course that mainly includes negative symptoms in SCZ and potential cognitive decline in these three SMI. Even though these disorders are defined as separated entities, their clinical boundaries remain unclear as they share common symptomatic and functional impairments. For example, abnormalities in neurocognitive functioning are associated with BD and SCZ [8]. Psychotic symptoms, such as delusions or hallucinations predominantly associated with SCZ, are also frequently experienced by patients during severe mood episodes belonging to BD or MDD [9]. These observed phenotypic similarities might be underpinned by shared brain alterations such as impairments in white and grey matter in BD and MDD [10]. SMI reduce patients’ life expectancy by 10 to 20 years [11], emphasizing the need for a better stratification of patients and identification of patients that are more likely to respond to a given treatment.

### 1.1. Pharmacogenetics Studies

The etiology of SCZ, BD and MDD disorders remains largely unknown, and numerous gene-specific or genome wide association studies have been conducted to investigate the possible genetic inheritance to these disorders [12,13]. However, conflicting results have been obtained, and many of the results could not be replicated, due to the complexity of the disease phenotypes, the implication of environmental factors in interaction with vulnerability genes and the polygenic nature of these disorders with more than 600 genes possibly involved in SCZ [12,13], more than 800 genes in BD [14,15] and more than 102 genes identified in MDD [16]. Some of these genes have been reported to be shared among these SMI in a meta-analysis [17]. Hence, pharmacogenetic studies have been used to identify genetic variants that are associated with treatment response in BD, SCZ and MDD. Several genetic variants have been identified, either in candidate gene approaches or in genome-wide association analyses (for review see [18,19,20]). This is consistent with the complexity of the response phenotypes and the polygenic nature of these SMI and raises the question of the transferability to clinical practice. Furthermore, transcriptomic modulations observed retrospectively or after the initiation of treatment in responders and non- (or poor) responders have been identified in BD, SCZ and MDD in animal and in vitro models, but also in patients [21,22,23,24,25]. Emerging evidences suggest that epigenetic marks could represent relevant biomarkers to be used as predictors of treatment response in several pathologies [26,27,28]. 

### 1.2. Epigenetic Mechanisms

Epigenetics is the study of mechanisms that control gene expression, irrespective of changes in the DNA sequence. Epigenetic mechanisms such as DNA methylation and histone modifications regulate chromatin and thereby the access of transcription factors. Another mechanism involves miRNAs (microRNAs) that target via seed sequences of single, or multiple, mRNAs leading to their down-regulation. All these mechanisms contribute to gene expression alterations, and they have been extensively reviewed [29,30]. These mechanisms can regulate the expression of several genes at the same time and for some of them (DNA methylation and miRNA) undergo transgenerational transmission [31]. Therefore, they may account for the polygenic nature, the partial heredity, and the differential gene expression modulations observed. DNA methylation is one of the most studied epigenetic mechanisms; it consists of the addition of methyl groups from *S*-adenylyl methionine (SAM) to the fifth carbon position of the cytosine residue in DNA by a family of DNA methyl transferase (DNMT) enzymes [29]. DNA methylation regulates gene expression through gene activation or gene silencing in the nervous system [32] and as a result, it may play a role not only in neurogenesis, but also in brain maturation and functioning [33,34,35]. 

### 1.3. Variability of Response to Treatments

In SMI, the treatment of acute phases and the long-term prevention of recurrence strategies mainly rely on four major classes of medications: atypical antipsychotics, mood stabilizers (anticonvulsants and lithium carbonate), and antidepressants. Long-term therapy with atypical antipsychotic drugs is the first line choice of treatment in SCZ. However, due to better tolerabilities and safety profiles, they may also be used in BD and MDD, for bipolar and unipolar depressions, but also for manic episodes [36,37]. Antipsychotics act mainly by binding to dopamine (DRD2) and serotonin (HTR2) receptors [38]. However, 30–40% of the patients with SCZ do not respond to the treatment in the acute phase and experience severe adverse effects [39]. Along with mood stabilizers such as lithium, antiepileptic drugs, valproate, lamotrigine, and carbamazepine, but also in combination with atypical antipsychotics quetiapine, olanzapine, and aripiprazole are used in BD management [40]. However, only 30% of the patients respond well in the chronic phase, and 70% show various degrees of treatment response in BD [41,42,43,44]. Tricyclic antidepressants, selective serotonin reuptake inhibitors (SSRIs), serotonin, and norepinephrine reuptake inhibitors (SNRIs) and mono amino oxidase inhibitors are the most commonly used medication in the treatment of depression. However, nearly 60% of the patients do not respond to the antidepressant therapy, and 30% do not respond at all [45,46]. 

In these three SMI, the lack of predictive biomarkers for treatment response sometimes results in a substantial proportion of patients experiencing potential adverse effects with ineffective therapies. Increasing data show that these drugs not only modulate DNA methylation in animal and cellular models, but also that the observed modulations can be associated with the response phenotypes in patients. Here, we will review the available data suggesting a role of DNA methylation in response to the treatment in three major psychiatric disorders: MDD, SCZ and BD. 

## 2. DNA Methylation Patterns in SCZ, BD and MDD

Multiple environmental factors have been shown to influence the pathogenesis of psychiatric disorders. Available preclinical and in-vitro studies indicate that altered epigenetic mechanisms such as DNA methylation, histone modifications, and miRNA regulation are associated with altered gene expression in major psychiatric disorders. Indeed, alterations of DNA methylation of genes important for the physiopathological aspects of SCZ, BD, and MDD, such as dopaminergic, serotoninergic, and Brain-Derived Neurotrophic Factor (BDNF) pathways have been reported [47,48,49,50]. In line with the shared genetic and environmental risks in SCZ and BD, hypomethylation of *FAM63B*, and an intergenic region on chromosome 16 have been proposed as common epigenetic risk factors in these two pathologies [51,52]. However, it is not possible to discriminate in these studies between the effects of the vulnerability to the disorders, the course of the disease, and those induced by the treatments. We concluded from the overall picture that the candidate gene, and the genome-wide approaches of DNA methylation patterns in SCZ, BD and MDD is of low contribution for the investigation of treatment response variability in these disorders. 

Therefore, in this review, we will focus on the reported effects of treatments on DNA methylation and their potential influence on the therapeutic response. Therapeutic strategies in SCZ, MDD and BD are generally based on similar classes of molecules (antidepressant, antipsychotics, and mood stabilizers) that are used in distinct dose and temporal combinations. Indeed, the lack of predictive markers for response lead to lengthy trial and error processes, and delayed optimal care of patients. For example, in SCZ, the current guidelines on the delay before evaluating the non-response to an antipsychotic and the switch for another differ substantially, but they are estimated at around two to three months [53,54,55]. In MDD, antidepressant acute response can be characterized after a mean delay of eight weeks; however, a recent meta-analysis found that their effects are stable over six months, as compared to the placebo [56]. In the case of BD, the characterization of lithium response can take up to two years to be characterized [57]. Interestingly though, several recent studies reported that these drugs can modulate epigenetic mechanisms at several levels of regulation. For instance, histone deacetylase 1 (*HDAC1*) was shown to be directly inhibited by valproic acid (VPA), while its expression can be downregulated by lithium [58,59]. Both mechanisms result in a decreased HDAC activity in cells. Similarly, the glutamatergic agonist LY379268-induced demethylation effects in *Reln*, *BDNF*, and *Gad67* genes may underlie antipsychotic effects in a mouse model [60]. These preclinical and clinical evidences indicate that pre-existing or psychotropic drug-induced epigenetic mechanisms may play a novel role in therapeutic response [61,62]. This review will focus on the most commonly studied epigenetic modification: DNA methylation, as a biomarker of treatment response in SCZ, BD and MDD. 

## 3. Treatment-Induced DNA Methylation Modifications in Animal Models

Results obtained from several preclinical investigations examining the changes of DNA methylation in response to antipsychotics, suggest that this epigenetic mark may play a role in the therapeutic response to these drugs (Table 1). Melka and colleagues showed that olanzapine could induce DNA methylation changes in dopamine receptors (*DRD1*, *DRD2*, and *DRD5*) and cadherin gene families, and that these modifications could be associated with an enhancement of the response (reduced stress-induced locomotor activity) in a rat model [63]. Another study, showed that olanzapine-induced DNA methylation changes of cadherin/pro-cadherin genes can impact the antipsychotic response (reduced stress-induced locomotor activity) in rats [64]. Another atypical antipsychotic, quetiapine, has been found to modulate DNA methylation at the promoter of *SLC64A* in SK-N-SH cells [65]. Furthermore, treatment with the typical neuroleptic haloperidol decreased the methyl cytosine (mC) content specifically in the brain tissue of female rats, and increased mC content specifically in the liver of male rats [66]. These latter results highlight the possible discrepancies between brain and peripheral DNA methylation modulations by drugs, as well as differences between males and females. Other drugs have also been shown to modulate DNA methylation in rodent model of SCZ (Table 1). Hence, VPA alone or in combination with atypical antipsychotic drugs induced demethylation effects of the *Reln* and *GAD67* promoters selectively in mouse brains [67]. Moreover, in line with the hypermethylation of *Reln* observed in the brain of patients with SCZ, imidazenil or VPA can reverse the hypermethylation of the *Reln* gene promoter in a mouse model of SCZ [68]. The discrepancies observed in blood vs. brain or female vs. male as well as the brain region specificities of DNA methylation observed in the rodent model will represent key issues to be considered for a transfer to bedside treatments.

As previously detailed, this has been tested in mice, and the observed demethylation of *GAD67* and *Reln* genes may also influence the therapeutic response in BD [67] (Table 1). In addition to modulations of histone acetylation, VPA also decreases DNA methylation at the *Reln* promoter in vitro [69,70,71]. Since the *BDNF* gene has been associated with the pathophysiology and symptoms of BD, its promoters have been the best studied. Incubation with lithium for 48 h was found to significantly decrease DNA methylation at the *BDNF* promoter IV in cultured rat hippocampal neurons; in addition, simultaneous increase of *BDNF* mRNA levels was also reported [72,73]. Fourteen days of treatment of mice with VPA induced a specific decrease of DNA methylation at the distal CpG island of the *Cdkn* p21 (cyclin dependent kinase inhibitor) promoter in the hippocampus, which could explain the observed increase in mRNA levels of this gene [74]. Similarly, the observed increase of *Glt1* (Glutamate transporter) transcript induced by VPA in rat primary astrocyte cell cultures could be attributed to several epigenetic changes, including a decrease of DNA methylation at the promoter of this gene [75]. Modulation of DNA methylation at imprinted loci have also been reported in stem cells after incubation with lithium at concentrations greater that the therapeutic range [76]. At therapeutic concentrations, lithium, carbamazepine, and VPA were found to modulate a large number of genes in SK-N-SH neuronal cells [77]. Most of the genes affected by carbamazepine and VPA were common, while lithium influences DNA methylation, not only for these genes, but also additional specific genes [77]. 

Antidepressants’ effects on DNA methylation have also been studied (Table 1). Administration of the SSRI escitalopram was associated with a decrease in DNA methylation at the *S100a10* (S100 Calcium Binding Protein A10) gene promoter region, and an increase of its transcripts in the prefrontal cortex in rat [78].

It is important to note that there are only a few studies published on cell lines or in animal models of depression. Several rodent models are available, and they could help in understanding the central epigenetic effects of antidepressants. However, the growing body of evidence in rodents and cell lines show that antipsychotics, antidepressants, and mood stabilizers influence DNA methylation at promoters of genes involved in their targeted pathways, drug metabolism, but also in various other cellular pathways. The role of these induced changes in treatment response have been assessed in cross-sectional and longitudinal human studies.

## 4. DNA Methylation Modifications and Responses to Treatment in Human Studies

### 4.1. Cross-Sectional Studies

- Schizophrenia: There is evidence that antipsychotics induce the alteration of the methylation status of several genes, including those coding for proteins and pathways targeted by these drugs. Very few human studies have investigated the influence of epigenetic mechanisms on the response to antipsychotics, but the DNA methylation status of candidate genes have been found to be differentially modulated by treatment in patients with SCZ according to their response phenotypes (Table 2). In a cohort of 177 SCZ patients, Melas and colleagues reported that patients treated with haloperidol (*n* = 16) displayed a significantly higher level of global DNA methylation in blood [49], as compared to other antipsychotic drugs. Interestingly though, correlations between the DNA methylation levels and the response status of SCZ patients have been found in several studies. However, a tendency to reverse hypermethylation at the *DTNBP1* (Dysbindin) gene promoter in post-mortem brain samples of schizophrenic patients was found with antipsychotic treatments [79].
- Bipolar disorder: The decreased DNA methylation of the *DTNBP1* gene promoter with antipsychotic drug treatment found in the post-mortem brain samples of patients with SCZ was not seen in post-mortem brains from patients with BD, probably due to small number of patients using classic antipsychotics [79] (Table 2). A lithium-induced decrease of global DNA methylation was found in lymphoblast cell lines derived from 14 lithium-responder BD patients, as compared to 16 healthy controls [80]. Recently, Houtepen and colleagues investigated the effects of antipsychotics (olanzapine and quetiapine) and mood stabilizers (lithium, VPA, carbamazepine) on genome-wide DNA methylation in blood samples from 172 patients with BD. After adjustment for drugs effects on blood cell types, composition-only VPA and quetiapine modified the DNA methylation status significantly [81]. In a study of global DNA methylation in the leukocytes of BD patients, no differences were found compared to the healthy control. However a significantly lower DNA methylation level was observed in patients on lithium monotherapy, compared to controls or BD patients treated with a combination of lithium + VPA [82]. In this study, the DNA methylation level could not be correlated with the lithium response as assessed with the Alda scale. However, since global DNA methylation studies provide an imprecise picture of the effect of a given drug, gene-specific effects have been investigated. A decrease of DNA methylation at the promoter I of *BDNF* was observed in the Peripheral Blood Mononuclear Cells (PBMC) of BD patients with antidepressant therapy, compared to no antidepressant therapy [50]. Similarly, patients treated with lithium or VPA displayed a decrease of DNA methylation levels, as compared to other medications [50,83]. A trend, yet not significant, for a decreased DNA methylation level at the *BDNF* promoter I was found in another study with a slightly different design [84].
- Major depressive disorder: A large study explored the DNA methylation of the *BDNF* gene in patients with MDD (*n* = 207), BD (*n* = 59), and controls (*n* = 278). They reported an increased methylation of *BDNF* gene in patients with MDD compared to those with BD and controls. Somewhat surprisingly, they also found that the increased methylation of *BDNF* is associated with antidepressant therapy, but not with the clinical features of MDD [85]. Although very informative, these studies did not discriminate between the antidepressant classes. The only available study on therapeutic response to the SSRI, paroxetine, reported an association with the methylation level of the *PPFIA4* (Protein Tyrosine Phosphatase, Receptor Type, F Polypeptide, Interacting Protein, Alpha 4) and *HS3ST1* (heparin sulfate-glucosamine 3-sulfotransferase 1) genes in MDD responders, compared to the worst responders (*n* = 10 per group) [86].

These data indicate that DNA methylation can be influenced by antipsychotics, mood stabilizers, and antidepressants in SCZ, BD and MDD patients. Moreover, these modulations of DNA methylation can be associated with a clinical response. However, these data obtained in retrospective studies do not allow for differentiation between pre-existing differences in DNA methylation (influenced by heredity and/or environmental factors) and those induced by the treatments. Longitudinal designs are therefore required in order to understand the role of DNA methylation in treatment responses, and their potential use as predictive or diagnostic biomarkers in these disorders.

### 4.2. Longitudinal Studies

- Schizophrenia: DNA methylation change at the 13th CpG site of *HTR1A* is associated with negative symptoms in patients with SCZ after 10 weeks of treatment with antipsychotic drugs (*n* = 82) [87] (Table 3). Likewise, clozapine-induced DNA methylation changes in the CREB-binding protein (*CREBBP*) gene are inversely correlated with the percentage of Positive and Negative Syndrome Scale (PANSS) changes in treatment-resistant SCZ patients (*n* = 21) [88]. A recent study in Chinese Han schizophrenic patients investigated not only genes that were involved in the dopaminergic and serotoninergic pathways, but also in the metabolism and transport of risperidone. They found no significant CpG sites in *HTR2A*, *ABCB1*, and *DRD2* gene promoters associated with responses, while differentially methylated CpG of the drug-metabolizing enzymes *CYP3A4* and *CYP2D6* genes promoter regions were associated with a response to risperidone [89]. Furthermore, a whole-genome study of DNA methylation modifications before and after treatment with antipsychotics found gender-specific differences in the methylation profiles of patients with SCZ. Significant differences were observed in the male patient group in complete remission [90]. In this study methylation levels of six genes (*APIS3*, *C16orf59*, *KCNK15*, *LOC146336*, *MGC16384* and *XRN2*) and nine genes (*C16orf70*, *CST3*, *DDRGK1*, *FA2H*, *FLJ30058*, *MFSD2B*, *RFX4*, *UBE2J1* and *ZNF311*) were respectively identified as good markers of treatment-induced effects, and good predictive markers of treatment response [90].
- Major depressive disorder: Several of the drugs used to treat an MDD target, a serotonin transporter, an association between its DNA methylation levels before treatment and impaired treatment response after 12 weeks of antidepressant therapy in patients with MDD (*n* = 108) have been reported [91] (Table 3). Another study comparing the methylation levels before and after six weeks of antidepressant therapy showed that an increased DNA methylation at the third CpG site of *SLC6A4* was associated with better therapeutic response in patients with MDD [92]. The results of Okada and colleagues were confirmed in a naturalistic study of MDD patients (*n* = 94) treated with escitalopram [93]. They found that higher methylation at the *SLC6A4* gene was associated with better treatment response after six weeks of treatment (Table 3). However, the response status to escitalopram was found not associated with the DNA methylation level of another gene *MAO-A* (mono amino oxidase A) in 61 MDD patients [94]. Recently, hypomethylation at two CpGs sites (*HTR1A* CpG 668 and *HTR1B* CpG 1401) was found to significantly differ in remitter and non-remitter Chinese Han patients with MDD (n = 85) with escitalopram treatment [95]. Very promising results have been obtained in MDD patients treated with escitalopram (*n* = 80) or with the tricyclic antidepressant nortriptyline (*n* = 33) [96]. In this study, higher DNA methylation level at the fourth CpG island of the interleukin-11 (*IL-11*) gene before treatment was associated with a better response to escitalopram, while hypomethylation at the same site was associated with a better nortriptyline response (Table 3). These results suggest that DNA methylation levels before treatment could be a predictor of the best suited antidepressant for an individual.

Few longitudinal studies have been published so far, and the results were obtained from peripheral samples. Nonetheless, they suggest that DNA methylation might be used as a predictive biomarker before the initiation of treatment or a monitoring biomarker of the efficacy of therapies in SCZ and MDD. Unfortunately, there is, for the moment, no available prospective study examining genome-wide DNA methylation modulation by specific mood stabilizers in BD patients. Further prospective studies are required to better understand how epigenetics could help physician in the prediction or the monitoring of treatment response in SCZ, BD and MDD.

## 5. Materials and Methods 

**Figure 1:**
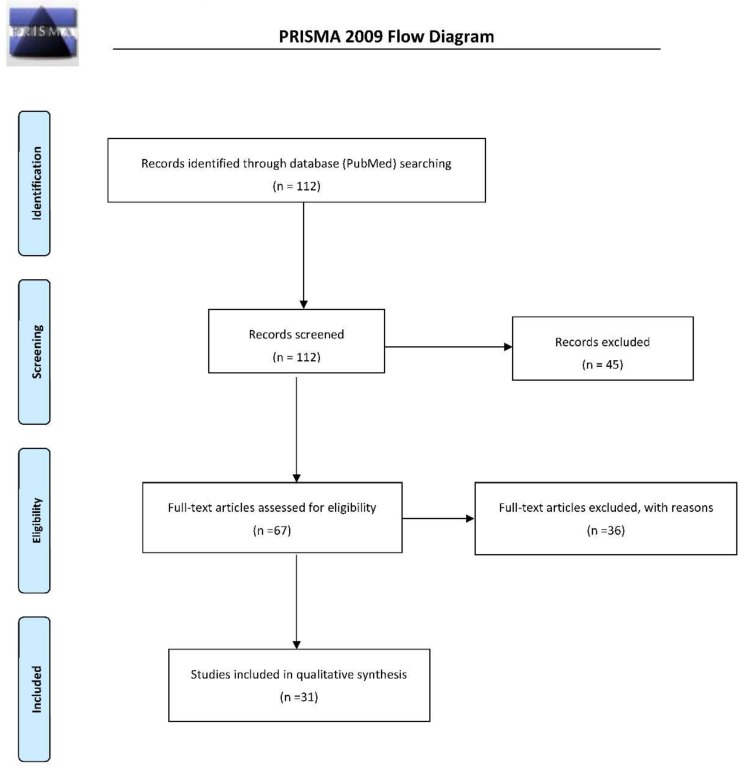
PRISMA (Preferred Reporting Items for Systematic Reviews and Meta-Analyses) flow-diagram of the screening strategy.

We conducted a literature search in the PubMed database until April 2018 using combinations of the following keywords: “DNA methylation”, “antipsychotic response”, “treatment response”, “bipolar disorder”, “major depressive disorder”, “schizophrenia”, “epigenetics”, and “antidepressants”. We followed the Preferred Reporting Items for Systematic Reviews and Meta-Analyses (PRISMA) guidelines [97]. Studies were included according to the following criteria: (a) being an original paper in a peer-reviewed journal; and (b) containing an epigenetic analysis of response treatment in BD, MDD and/or SCZ samples. Figure 1 summarizes the search strategy used for selecting the studies (identification, screening, eligibility, inclusion process) in the present review. Two blinded independent researchers (GAC and CMC) conducted a two-step literature search. Discrepancies were resolved by consultations with the other authors. The reference lists of the articles were also manually checked for additional relevant studies. We identified 112 articles on the basis of their titles; 67 abstracts focused on DNA methylation as a biomarker of treatment response in cellular or rodent models, or in BD, SCZ and/or MDD patients were selected. Exclusion criteria included (i) studies not written in English; (ii) review articles, book chapters, conference abstracts, and case studies; (iii) studies not related to response to treatment. The final selection consisted of 31 original articles specifically related to DNA methylation in treatment responses to BD, SCZ and MDD. This systematic review will first present an overview of the DNA methylation and its role in gene expression and treatment response. We will then summarize studies on DNA methylation patterns in SCZ, BD and MDD as results of potential interest for the investigation of treatment response variability. Then, we will present treatment-induced DNA methylation modifications in animal models and DNA methylation modifications in response to psychotropic treatments in human studies. Finally, the overall picture will be discussed. 

## 6. Conclusions

The characterization of biomarkers throughout the disease course is a key element, not only for understanding its pathophysiology, but also to monitor treatment responses. Case control studies have shown differential DNA methylation levels in patients, as compared to control subjects. Recent data in high-risk offspring from BD patients suggest that the observed differences in DNA methylation are directly related to the familial environment [98]. Likewise, the significant decreased methylation of the CpG site at the −1438A/G polymorphism site of the *HTR2A* (5-hydroxytryptamine receptor 2A) receptor observed in SCZ and BD patients’ saliva, but also in their first degree relatives, suggests an effect of environmental factors independent of the onset and course of the disorders, as well as the treatments [99]. The course of the disease might also influence the DNA methylation levels, as suggested by the accelerated epigenetic aging observed in older BD patients as compared to control subjects, while no significant difference was observed in younger patients [100]. Despite this very complex landscape, available evidence supports the hypothesis of the DNA methylation role in therapeutic response to antipsychotics, mood stabilizers, and antidepressants in the treatment of SMI, such as SCZ, BD and MDD. Finding novel pathways by targeting the epigenome in the context of a treatment response may also help to understand the role of these pathways in diseases. These pathways might also be targeted by drug repurposing strategies in order to identify already known medications that could alter the identified epigenetic marks [101]. Finally, this may also help clinicians to specifically prescribe psychoactive treatments targeting the identified biological pathways to move towards a more tailored and personalized strategy of medicine in psychiatry. However, findings from in vitro and in vivo models studies suggesting the DNA methylation as a therapeutic target may not be relevant to human subjects. Notably, gender specificities and appropriate sample plan designs are key issues for transferability to clinical practice. SMI often require a complex mixture of several co-medications to improve patients’ conditions. However, the main limitations of this review is the fact that only a limited number of human studies are available for the moment, and they target the effects of one type of drug, or even one specific drug at a time, whereas patients often receive polypharmacy. Therefore, the combined effects of antidepressants, antipsychotics, and mood stabilizers are not taken into account. The reported epigenetic effects might not represent real life. Moreover, as a result of the cross-sectional design of most studies it cannot be concluded that the epigenetics marks observed in presence of a given medication are solely the effect of the drug, and not the sum of the effects of drugs and other environmental factors that are likely to modify DNA methylation. Very few studies have been reported in human subjects, and the use of several co-medications, retrospective design, and various response evaluation time points represent a serious limitation. Further studies with monotherapy treatment, prospective study designs considering the duration of the response assessment, co-medications, and addressing the environmental factors during the illness as well as treatment, will better explain the DNA methylation signatures. These studies will be useful in the implementation and the development of efficient and personalized therapeutic strategies. 

## Figures and Tables

**Table 1:** Animal and cellular studies.

Model/Tissue	Method	Main Findings	Reference
Leukocytes/brain/liver tissues of rat	High-performance liquid chromatography	• Decreased mC in the brain of haloperidol-treated female rats Increased mC in liver DNA in haloperidol-treated male rats	[66]
Frontal cortex from a mouse model receiving l-methionine	Bisulfite conversion + PCR + sequencing	• VPA corrects *Reln* promoter hypermethylation induced in this SCZ-like model	[68]
Brian/liver tissues of rats	MeDIP	• 19 days of treatment with olanzapine increases DMA methylation at several dopaminergic genes	[63]
Mouse prefrontal cortex and striatum	MeDIP ChIP followed by qPCR	• VPA, clozapine, sulpiride, VPA + clozapine, and VPA + sulpiride treatment induce DNA demethylation of *GAD67* and *Reln* genes	[67]
Human neuroblastoma cell lines SK-N-SH	Infinium HumanMethylation27 BeadChip + bisulfite sequencing	• Quetiapine decreases DNA methylation of the CpG3 island of *SLC6A4*	[65]
Brian/liver tissues of rats	MeDIP	• Olanzapine alters DNA methylation at several cadherin/procadherin promoter region	[64]
Cultured rat hippocampal neurons	Methylation-specific PCR	• 48 h exposition to 1 and 2 mM lithium: 0.6-fold decrease DNA methylation at the promoter IV of BDNF • 2-fold increase of mRNA	[73]
Mouse hippocampal cells	PCR methylation-sensitive restriction site analysis	• 30% decrease of DNA methylation at the distal CpG island of the Cdkn p21 gene	[74]
Rat primary astrocytes	luminometric methylation analysis (LUMA)	• Decrease of DNA methylation at the Glt-1 promoter	[75]
Mouse embryonic and neural stem cells	Bisulfite sequencing	• 10 and 20 mM of lithium induce a 66% decrease of DNA methylation at the *Igf2/H19* differentially methylated domain (DMD) in embryonic stem cells • 5 mM of lithium induce a 33% decrease of DNA methylation at the *Igf2/H19* DMD in neural stem cells	[76]
Human neuroblastoma cell lines SK-N-SH	Infinium HumanMethylation27 BeadChip	• Hypermethylation of 345 genes (lithium), 64 genes (VPA), and 64 genes (carbamazepine) • Hypomethylation of 138 genes (lithium), 36 genes (VPA), and 14 genes (carbamazepine)	[77]

Polymerase Chain reaction (PCR), quantitative PCR (qPCR), Methylated DNA Immunoprecipitation (MeDIP), Chromatin ImmunoPrecipitation (ChIP).

**Table 2:** Cross-sectional studies.

Model/Tissue	Method	Main Findings	Reference
Leukocytes of SCZ patients (*n* = 177) and controls (*n* = 171)	luminometric methylation analysis (LUMA)	• Increased global methylation in patients treated with haloperidol compared to other treatments	[49]
Saliva samples of SCZ (*n* = 30), first-degree relatives of SCZ (*n* = 15), controls (*n* = 30)/postmortem brain samples of patients with SCZ (*n* = 35) and BD (*n* = 35)	Quantitative methylation specific PCR	• Increased DNA methylation of DTNBP1 promoter in the saliva of patients with SCZ compared to controls • Inverse correlation between DTNBP1 methylation and expression in post-mortem brains of SCZ patients • Trend to reduced DNA methylation of DTNBP1 by antipsychotics treatment	[79]
Postmortem brain samples of patients with BD (*n* = 35) and controls (*n* = 35)	Quantitative methylation specific PCR	• No significant difference of the *DTNBP1* promoter region associated with antipsychotic treatment in patients with BD	[79]
Transformed lymphoblast cell lines from: lithium responders BD (*n* = 14), affected relatives (*n* = 14), unaffected relatives (*n* = 16), Healthy controls (*n* = 16)	ELISA	• Decreased DNA methylation in cell lines of BD patients, affected and unaffected relatives, compared to healthy controls • Lithium-induced decrease in global DNA methylation in BD patients (lithium responders) compared to controls	[80]
Whole blood of patients with BD (*n* = 172), Human	Infinium Human-Methylation450 BeadChip	• Quetiapine, VPA showed significant DNA methylation alterations patients with BD	[81]
Peripheral blood from BD patients on Li monotherapy (*n* = 29), Lithium + VPA (*n* = 11), Lithium + antipsychotics (*n* = 21), healthy controls (*n* = 26)	ELISA	• Hypomethylation of DNA in BD patients treated with lithium monotherapy vs. lithium + VPA or healthy controls • No significant relation between DNA methylation and lithium response	[82]
PBMC from BDI (*n* = 45), BDII (*n* = 49), and control subjects (*n* = 52)	Methylation-specific qPCR	• Decrease of DNA methylation at *BDNF* promoter I in patients with antidepressant therapy vs. controls • Decrease of DNA methylation at *BDNF* promoter I in patients with lithium therapy vs. other medications • Decrease of DNA methylation at *BDNF* promoter I in patients with VPA therapy vs. other medications	[50]
PBMC from BDI (*n* = 45), BDII (*n* = 45)	methylation specific qPCR	• Decrease of DNA methylation at *PDYN* promoter I in patients with lithium or VPA therapy (*n* = 25) vs. other medications	[83]
PBMC from BDI (*n* = 61), BDII (*n* = 50), MDD (*n* = 43) patients	methylation specific qPCR	• Not significant trend for a decrease of DNA methylation in patients treated with lithium and VPA	[84]
PBMC from MDD (*n* = 207), BD (*n* = 59) and controls (*n* = 278)	Methylation-specific quantitative PCR	• *BDNF* gene exon I promoter methylation increased in MDD compared to BD and controls • Increased *BDNF* DNA methylation in MDD patients associated with antidepressant therapy	[85]
Peripheral leukocytes from MDD patients (10 best responders and 10 worst responders to paroxetine)	Infinium Human-Methylation450 BeadChip	• Methylation levels of the CpG sites in *PPFIA4* and *HS3ST1* gene can discriminate between best and worst responders	[86]

ELISA (Enzyme-Linked Immunosorbent Assay), Polymerase Chain reaction (PCR), quantitative PCR (qPCR), Bipolar disorder type 1 (BDI), Bipolar disorder type 2 (BDII).

**Table 3:** Longitudinal studies.

Model/Tissue	Method	Main Findings	Reference
Peripheral blood of patients with SCZ (*n* = 82)	Bisulfite conversion + PCR + pyrosequencing	• Decreased DNA methylation at CpG13 of *HTR1A* associated with poorer response to antipsychotics	[87]
Peripheral blood from SCZ patients (*n* = 21)	Infinium Human-Methylation450 BeadChip	• Clozapine-induced DNA methylation changes in the *CREBBP* gene were significantly correlated with clinical improvements	[88]
Peripheral blood from SCZ patients; good responders (*n* = 88), poor responders (*n* = 54)	Methylation-specific PCR + mass spectrometry	• Seven CpGs at *CYP3A4* and *CYP2D6* genes were differentially methylated in good vs. poor responders to risperidone therapy	[89]
Peripheral blood from SCZ patients *n* = 20 (12 M/8 F)	MeDIP ChIP	• Before treatment: nine genes with DMR in male SCZ patients in complete remission after treatment (vs. matched control subjects) • After treatment: six genes with DMR in male SCZ patients in complete remission after treatment (vs. matched control subjects) • Before treatment: one gene (M1R181C) with DMR in female SCZ patients in complete remission after treatment (vs. matched control subjects) • After treatment: one gene (BCOR) with DMR in female SCZ patients in complete remission after treatment (vs. matched control subjects)	[90]
Leukocytes from patients with MDD (*n* = 108)	Bisulfite conversion followed by PCR	• Increased of SLC6A4 DNA methylation level associated with impaired treatment response to antidepressants	[91]
Whole blood from patients with MDD (*n* = 50 before treatment and *n* = 40 after 6 weeks of treatment) and controls (*n* = 50)	Methylation-specific PCR + mass spectrometry	• Methylation level of the third CpG site of SLC6A4 gene association with better therapeutic response to antidepressant therapy in patients MD	[92]
Whole blood from patients with MDD (*n* = 94), (Human)	Bisulfite conversion + PCR	• Increased DNA methylation of *SLC6A4* gene associated with better treatment response to escitalopram	[93]
Whole blood from patients with MDD, (*n* = 61) (Human)	Bisulfite conversion + PCR	• No major influence of mono amino oxidase (MAO-A) gene methylation status on escitalopram response	[94]
Peripheral blood of patients with MDD (*n* = 85)	Bisulfite sequencing	• Significant association of 668 CpG sites of HTR1A and 1401 CpG sites of HTR1B gene methylation with treatment response to escitalopram	[95]
Peripheral blood samples of patients with MDD treated with escitalopram (*n* = 80) or nortriptyline (*n* = 33)	Bisulfite conversion + PCR	• Fourth CpG island hypomethylation of IL-11 gene associated with better response to nortriptyline • Hypermethylation of fourth CpG island of IL-11 gene associated with better response to escitalopram	[96]

Polymerase Chain reaction (PCR), quantitative PCR (qPCR), Methylated DNA Immunoprecipitation (MeDIP), Chromatin ImmunoPrecipitation (ChIP).

## Abbreviations

SMI	Severe mental illnesses
SCZ	Schizophrenia
BD	Bipolar disorder
MDD	Major depressive disorder
